# Antihelminthic benzimidazoles potentiate navitoclax (ABT-263) activity by inducing Noxa-dependent apoptosis in non-small cell lung cancer (NSCLC) cell lines

**DOI:** 10.1186/s12935-014-0151-3

**Published:** 2015-02-04

**Authors:** Lloyd T Lam, Haichao Zhang, John Xue, Joel D Leverson, Anahita Bhathena

**Affiliations:** Oncology Research, Abbvie, 1 North Waukegan Road, North Chicago, IL 60064-6098 USA; AbbVie, Tumor Genomics, bldg AP-10, dept R4CD, AbbVie, 1 North Waukegan Road, North Chicago, IL 60064-6098 USA

**Keywords:** Benzimidazoles, Navitoclax, Caspase, Apoptosis

## Abstract

**Background:**

Evasion of apoptosis is a hallmark of cancer cells. One mechanism to deregulate the apoptotic pathway is by upregulation of the anti-apoptotic Bcl-2 family members. Navitoclax (ABT-263) is a Bcl-2/Bcl-x_L_ inhibitor that restores the ability of cancer cells to undergo apoptosis.

**Methods:**

In this study we performed a high-throughput screen with 640 FDA-approved drugs to identify potential therapeutic combinations with navitoclax in a non-small cell lung cancer (NSCLC) cell line.

**Results:**

Other than a panel of cancer compounds such as doxorubicin, camptothecin, and docetaxel, four antihelminthic compounds (benzimidazoles) potentiated navitoclax activity. Treatment with benzimidazoles led to induction of the pro-apoptotic protein Noxa at the mRNA and protein level. Noxa binds and antagonizes antiapoptotic protein Mcl-1. siRNA-mediated knock-down of Noxa completely rescued benzimidazole-potentiated navitoclax activity. In addition, inhibiting caspase 3 and 9 partially rescued benzimidazole-potentiated navitoclax activity.

**Conclusions:**

We have identified compounds and mechanisms which potentiate navitoclax activity in lung cancer cell lines. Further validation of the benzimidazole-potentiated navitoclax effect *in vivo* is required to evaluate the potential for translating this observation into clinical benefit.

**Electronic supplementary material:**

The online version of this article (doi:10.1186/s12935-014-0151-3) contains supplementary material, which is available to authorized users.

## Background

Evasion of apoptosis is a hallmark of cancer cells [1]. One mechanism of apoptotic pathway deregulation is via upregulation of the anti-apoptotic Bcl-2 family members [2]. Apoptotic pathway proteins belong to a family of BH-domain-containing proteins comprising three classes [3]: 1) multi-domain anti-apoptotic (Bcl-2, Bcl-x_L_, Bcl-w, Bfl-1/A1, and Mcl-1), 2) multi-domain pro-apoptotic (Bax, Bak), and 3) BH3-only pro-apoptotic (Bid, Bim, Bad, Bik, Noxa, Puma, Bmf, and Hrk). The BH3-only proteins contain a single BH3 domain that interacts with specific anti-apoptotic proteins [4]. For example, Bcl-2 and Bcl-x_L_ bind Bad but not Noxa. By contrast, Mcl-1 and A1 bind Noxa but not Bad protein. Other BH3 domain proteins such as Bim and Puma are bound by all anti-apoptotic proteins. Bax and Bak are the “effectors”. Once activated, they form complexes and permeabilize the outer mitochondrial membrane, resulting in the release of cytochrome c and other pro-apoptogenic proteins to induce the cell death pathway.

Navitoclax (ABT-263) is a first-in-class Bcl-2 and Bcl-x_L_ antagonist that restores the ability of cancer cells to undergo apoptosis [5]. It exhibits potent activity as a single agent against several tumor types including small cell lung cancer (SCLC) and hematological malignancies [6]. Resistance to navitoclax in most solid tumors is due to its low affinity to Mcl-1 [7,8]. In this regard, it was shown that Mcl-1 could contribute to the overall resistance of SCLC and other cancer cell lines to ABT-737, an analog of ABT-263 [7-10]. Thus, combining agents that target Mcl-1 with ABT-737 might restore the apoptotic program in cells expressing high levels of Bcl-x_L_ and Mcl-1 [11].

Multiple targeted agents have been shown to increase the efficacy of ABT-737 through the modulation of Bcl-2 family proteins. One of the best known examples is CDK inhibitors, which strongly decrease Mcl-1 expression and overcome Mcl-1-dependent resistance to ABT-737 [8]. Sorafenib has been shown to reduce Mcl-1 in hepatoma cells in combination with ABT-737 [12]. Similarly, synergistic induction of apoptosis by ABT-737 and imatinib mesylate was observed in gastrointestinal stromal tumor cells [13]. Interestingly, EGFR inhibitors were shown to combine with ABT-737 by inducing Bim [14,15]. Bim also plays a role in combination of MEK inhibitors and ABT-263 [16,17]. Finally, bortezomib potentiates ABT-737 activity by inducing Noxa, a BH3-only protein [18].

In this study we performed a high-throughput screen with 640 FDA-approved drugs to elucidate mechanisms for potentiation of navitoclax activity and to identify potential therapeutic combinations. We reasoned that these compounds have already undergone stringent testing for safety and toxicity and could therefore accelerate the process for clinical testing. From the screen, we identified a class of antihelminthic compounds as potentiators of navitoclax activity. Furthermore, we showed that the mechanism of potentiation is via induction of Noxa. Our results identify a novel combination regimen strategy for improving navitoclax activity and support a viable hypothesis for additional testing to assess the potential benefit of this novel combination.

## Results

### Benzimidazoles identified as potentiators of navitoclax activity

**Figure 1:**
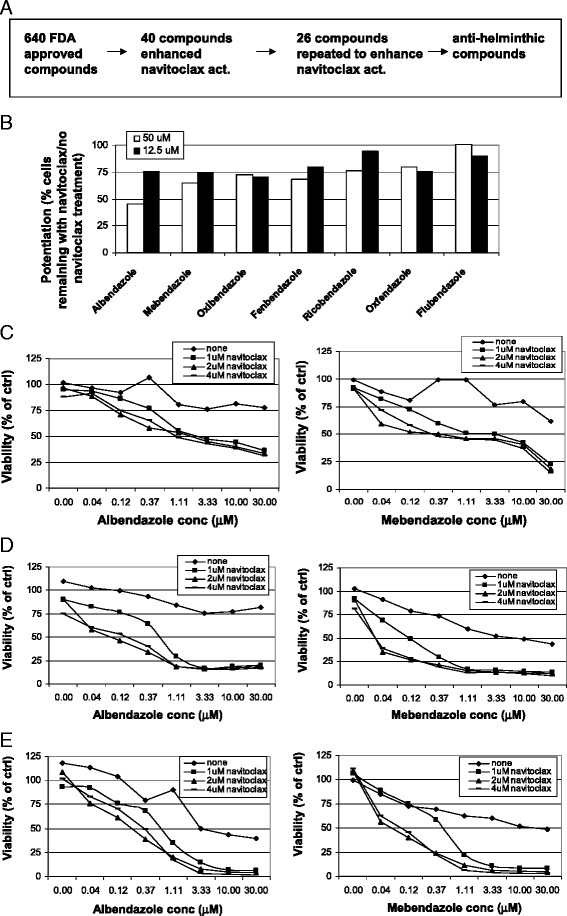
**High-throughput screening of a FDA approved drug library identifies a class of antihelminthic benzimidazoles compounds that potentiate the effect of navitoclax in NSCLC cell lines. (A)** Schematic representation of high-throughput FDA approved compound library screen. **(B)** Hcc827 cells were treated with the FDA approved drug library (640 compounds) in the presence or absence of 1 μM navitoclax. Four dilutions per compound (0.78, 3.13, 12.5, and 50 μM) were tested. Of the 40 compounds from the initial screen, 26 were validated and a class of antihelminthic benzimidazoles was chosen for further studies. Potentiation of navitoclax activity was calculated by % of cells remaining in the presence of these compounds plus navitoclax vs.% of cells remaining in the presence of these compounds alone. Validating anti-helminthic benzimidazoles potentiate navitoclax effect in three NSCLC cell lines. **(C)** Hcc827, **(D)** H292, and **(E)** H1993 cells were treated with increasing concentrations of albendazole or mebendazole in the presence of navitoclax (0, 1, 2, and 4 μM). Viability was determined after 1 day. Data shown are representative of at least two independent experiments.

Navitoclax is a first-in-class Bcl-2 inhibitor with demonstrated antitumor activity against SCLC solid tumor cell lines and hematologic malignancies such as chronic lymphocytic leukemia (CLL) and other leukemias [7,19]. However, resistance to this compound has been described [7,8]. To identify potential combination treatment regimens with navitoclax, we screened a library of 640 FDA-approved compounds in a NSCLC cell line, Hcc827, previously shown to depend on both Bcl-x_L_ and Mcl-1 for survival [20]. Hcc827 cells were treated with the library in the presence or absence of 1 μM navitoclax for 1 or 3 days (Figure 1A). Since the compounds that potentiated navitoclax activity identified from the 1- or 3-day screens were very similar, we set a cut off of >25% cell death after co-dosing with navitoclax for hits. Our initial screen identified 40 compounds that could potentiate the effect of navitoclax. Of these, 18 are known cancer chemotherapeutic agents (*e.g.*, doxorubicin, camptothecin, and docetaxel), consistent with results reported recently [21]. Subsequent repeats confirmed 26 compounds as potentiators of navitoclax activity. Since 4 of 7 benzimidazoles belonging to a class of antihelminthic compounds (albendazole, mebendazole, oxibendazole, and oxfendazole) were found to potentiate navitoclax activity in Hcc827 cells (Figure 1B), we decided to study the activity of these compounds further. Similar trends were observed in another NSCLC cell line, H292 (Additional file 1: Figure S1). Albendazole and mebendazole showed the strongest potentiation (Figure 1B). In addition, these agents have been used clinically to treat human alveolar echinococcosis, a lethal pulmonary helminthic infection, with minimal side effects to the host [22]. We therefore focused our studies further on these two compounds.

Benzimidazole monotherapy and combination with navitoclax was assessed in three NSCLC cell lines, Hcc827, H292 and H1993. Increasing concentrations of albendazole or mebendazole and navitoclax were used to treat these lines and viability was measured. Although these compounds show toxicity against these cell lines (up to around 50% at 10 μM), potentially due to their activity against the tubulins (see Discussion), they were more active when combined with navitoclax (Figure 1C, D, and E). The EC_50_ for both albendazole and mebendazole shifted from ≥30 μM to 1.11 μM in Hcc827 cells, and ~10 μM to ≤0.5 μM in H292 and H1993 cells. Bliss analysis showed strong synergism (Bliss score > 30) between albendazole or mebendazole and navitoclax in all three lines, suggesting these compounds strongly potentiated the activity of navitoclax.

### Noxa plays an important role in albendazole and mebendazole-induced navitoclax activity in NSCLC cell lines

**Figure 2:**
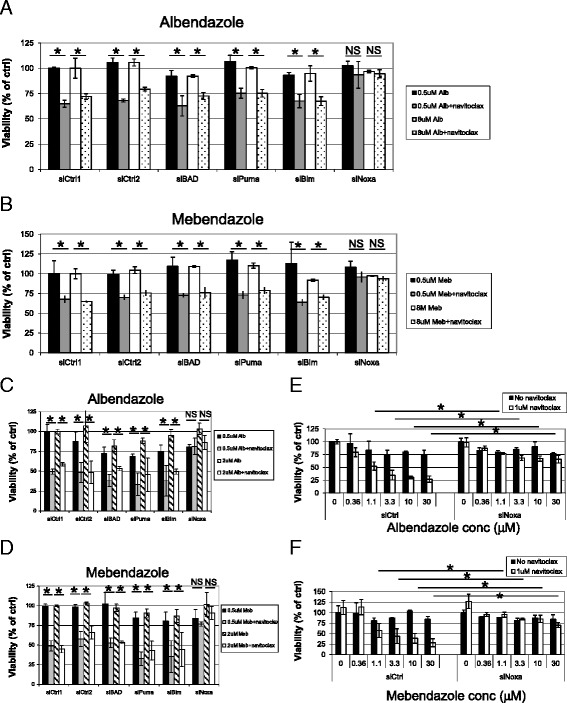
**Benzimidazoles mediate navitoclax activity through Noxa in Hcc827 and H292 cells.** Hcc827 cells were transfected with siRNA against four BH3 domain proteins for 1 day before adding **(A)** albendazole (0.5 or 8 μM) or **(B)** mebendazole (0.5 or 8 μM) +/− 1 μM navitoclax. Viability was determined after 1 day. H292 cells were transfected with siRNA against four pro-apoptotic BH3 domain proteins for 1 day before adding **(C)** albendazole (0.5 or 2 μM) or **(D)** mebendazole (0.5 or 2 μM) +/− navitoclax. Viability was determined after 1 day. H292 cells were transfected with siRNA against Noxa for 1 day before adding **(E)** albendazole (0 to 30 μM) or **(F)** mebendazole (0 to 30 μM) +/− navitoclax. Viability was determined after 1 day. *T*-test was carried out to test the significance of the observed differences between 2 conditions. *P < 0.05.

To identify the potential mechanisms whereby albendazole and mebendazole potentiate navitoclax activity, we utilized a small-scale functional genomics approach in which multiple proapoptotic BH3 domain proteins (Puma, Bad, Noxa, and Bim) were knocked down using siRNAs [23]. siRNAs that rescued the cells from the toxicity of albendazole or mebendazole and navitoclax combinations could serve to identify proteins involved in the underlying mechanism. We first tested the effect of these siRNAs in rescuing the potentiation effect of albendazole or mebendazole in Hcc827 cells. The levels of knockdown of Bim and Noxa by siRNA are shown in Additional file 2: Figure S2. Interestingly, only the silencing of Noxa led to a complete rescue of Hcc827 cells from benzimadazole-navitoclax combinations (Figure 2A and B). We confirmed this finding in H292 cells (Figure 2C and D). These results implicate Noxa as playing an important role in the benzidimazoles’ potentiation of navitoclax activity.

To assess whether Noxa is the sole mechanism for albendazole- and mebendazole- potentiation of navitoclax activity, we performed a titration experiment in which increasing concentrations of albendazole and mebendazole were used in the presence or absence of Noxa siRNA and navitoclax. In the presence of the control siRNA and navitoclax, potentiation was produced by both albendazole and mebendazole (Figure 2E and F). In contrast, in the presence of the Noxa siRNA and navitoclax, the potentiation was completely rescued even at pharmacologic concentrations of these compounds. These results further suggest that Noxa is the major mechanism mediating albendazole and mebendazole potentiation of navitoclax activity.

**Figure 3:**
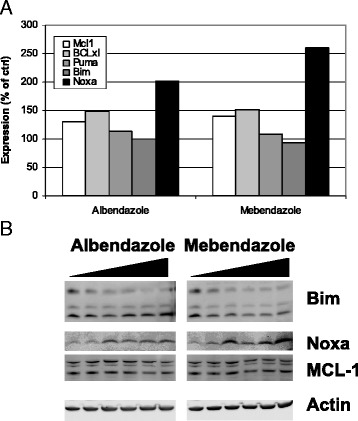
**Induction of Noxa mRNA and protein in H292 cells treated with albendazole and mebendazole. (A)** H292 cells were treated with or without 10 μM albendazole or mebendazole for 1 day. Cell lysate was prepared and Noxa, Bim, Puma, Mcl-1, Bcl-x_L_, and HPRT mRNA was measured using a branched DNA assay. The expression of these genes was normalized to HPRT control and no treatment control. **(B)** H292 cells were treated with increasing concentrations (0–10 μl) of albendazole and mebendazole for 2 days. Cell lysate was prepared and resolved on a 12% SDS polyacrylamide gel and probed with anti-Bim, anti-Noxa, and anti-Mcl-1. Antibody against actin was used as loading control. Data shown are representative of two independent experiments.

An independent evaluation of the role of Noxa to potentiate navitoclax activity was performed by assessing the induction of Noxa mRNA and protein expression upon albendazole and mebendazole treatment. We used a multiple branched DNA technique to measure the gene expression of Noxa, Bim, Puma, Mcl-1, and Bcl-x_L_ in H292 cells treated with albendazole and mebendazole. This sandwich nucleic acid hybridization assay can amplify the signal without requiring enzymatic amplification of the target RNA [24]. Only Noxa mRNA was induced upon albendazole and mebendazole treatment (Figure 3A). In addition, albendazole and mebendazole treatment leads to induction of Noxa protein (Figure 3B). These data are consistent with the rescue studies in Figure 2, in which Noxa is the major mechanism in albendazole and mebendazole potentiation of navitoclax activity.

**Figure 4:**
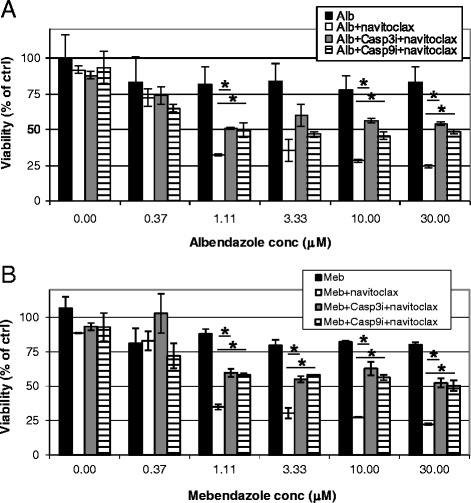
**Inhibitors of caspase 3 and 9 partially rescue benzimidazole-mediated navitoclax activity in H292 cells.** H292 cells were pretreated with caspase 3 and 9 inhibitors (50 μM) before adding **(A)** albendazole (0 to 30 μM) or **(B)** mebendazole (0 to 30 μM) +/− 0.5 μM navitoclax. Viability was determined after 1 day. *T*-test was carried out to test the significance of the observed differences between 2 conditions. *P < 0.05.

Caspases are proteases that “execute” apoptosis. To determine whether caspase 3 and caspase 9 are involved in the benzimidazoles’ potentiation of navitoclax activity, we pre-incubated H292 cells with inhibitors to caspase 3 and caspase 9 prior to treatment with albendazole or mebendazole and navitoclax. Figure 4 shows that both inhibitors of caspase 3 and caspase 9 partially rescued H292 cells from albendazole or mebendazole and navitoclax-induced toxicity. These data suggested that caspase 3 and caspase 9 mediate albendazole and mebendazole potentiation of navitoclax activity.

### Mebendazole potentiates navitoclax killing in multiple NSCLC cell lines

**Figure 5:**
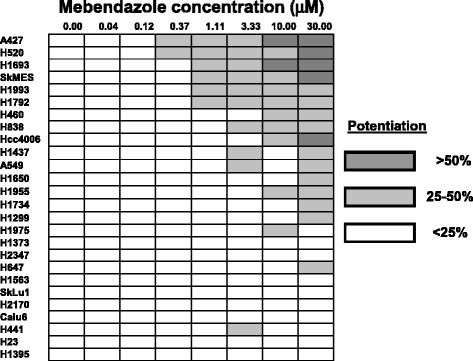
**Mebendazole potentiates navitoclax activity in multiple NSCLC cell lines.** 26 NSCLC cell lines were treated with increasing concentrations of mebendazole in the presence or absence of 1 μM navitoclax. Viability was determined after 1 day. Data are presented as % potentiation in the presence of navitoclax.

To expand our studies, we determined whether mebendazole can potentiate the effect of navitoclax in a larger panel of NSCLC cell lines. Of the 26 NSCLC cell lines tested, we found that potentiation was observed in 15 lines (with at least two concentrations having >20% cell death and one concentration having >25% cell death) (Figure 5). We have tested cell lines of other tumor origins and found that multiple lines (*e.g.*, MDA-MB-231, Hcc1806, Ovcar4, and PC3) show strong synergistic responses to albendazole with navitoclax (Additional file 3: Figure S3). Our findings suggest that combination with albendazole and mebendazole may enhance activity of navitoclax.

## Discussion

We report the identification of a class of benzidimidazole antihelminthic compounds (albendazole, mebendazole, oxibendazole, and oxfendazole) which markedly potentiate the activity of navitoclax in multiple NSCLC cell lines. In addition, we provide evidence that the mechanism for potentiation is through the induction of Noxa. Silencing the expression of Noxa completely rescues the cells from the effect of these combinations.

Benzimidazole compounds are commonly used and have broad spectrum antihelminthic activity against roundworms, tapeworms and flukes in animals and humans. Their major activity is through binding to parasite-tubulin, leading to inhibition of polymerization or assembly into microtubules. Interestingly, recent studies suggested that these compounds could be used in cancer treatments [25-27]. The mechanism of anti-tumor activity is through the binding of tubulin [28,29]. A phase I clinical trial using albendazole showed that the compound was well tolerated and the maximum tolerated dose was 2,400 mg per day [30]. Myelosuppression was the main dose-limiting toxicity. Fatigue and mild gastrointestinal upset were the other major adverse effects. Another study using a different antihelminthic, flubendazole, shows that inhibition of microtubule function occurs through a mechanism distinct from Vinca alkaloids and displays preclinical activity in leukemia and myeloma [31], suggesting these compounds may also be combined with classical antitubulins in cancer treatment. Potential problems with developing the antihelminthic compounds for cancer therapy include the rapid metabolism of these compounds in the liver [30].

We chose to employ the FDA-approved drug library to screen for potentiators of navitoclax activity. Our rationale included the following elements: 1) these compounds have already been tested for safety and efficacy and are approved for use in humans; 2) the use of compounds with good drug-like properties could accelerate the process of clinical testing; and 3) such a screen may reveal novel activities of these compounds that would not be identified otherwise. Indeed, our studies revealed an unknown mechanism of the antihelminthic compounds against cancer cells potentially other than binding to tubulin. In particular, we found that antihelminthic compounds can induce the expression of Noxa and thereby potentiate the activity of navitoclax.

Multiple studies have described the role of Noxa regulation in determining sensitivity to ABT-737 with or without modulation of Mcl-1 [7,18,32-38]. Agents that induce Noxa include chemotherapeutic agents such as etoposide, vinblastine, paclitaxel, dacarbazine, camtothecin, cisplatin, and targeted agents such as bortezomib. Since these agents represent different subclasses of cancer agents, it is likely that multiple mechanisms are involved in regulating Noxa. Earlier studies suggested that p53 is involved in Noxa regulation in response to DNA damage [39]. However, cells lacking p53 can activate Noxa in response to bortezomib [40,41]. In this case Noxa expression is induced via Myc [40]. In another study, the endoplasmic reticulum (ER)-associated protein degradation (ERAD) inhibitor Eeyarestatin I and bortezomib activate Noxa via eliciting a stress response program that involves the activation of transcription factors ATF3 and ATF4, which bind to the Noxa promoter. In addition, these agents block ubiquitination of histone H2A to relieve its inhibition on Noxa transcription [42]. A recent study showed that BH3 mimetics other than ABT-737 also antagonize Mcl-1 by activating the ER stress response, inducing ATF3, ATF4, and Noxa, which can then sequester Mcl-1 and inhibit its function [43]. How Noxa is regulated by the benzimidazoles is not known. We observed no correlation between p53 mutation status and sensitivity to benzimidazole-navitoclax combinations, suggesting that p53 does not regulate Noxa in response to benzimidazoles (Additional file 4: Figure S4). Treatment with these agents did not alter the expression of ATF3 or ATF4 in H292 and Hcc827 cells, suggesting that induction of Noxa is not a response to ER stress (data not shown). Further studies will be required to dissect the mechanisms of Noxa induction in these cells. It would be interesting to determine whether the binding of benzimidazoles to tubulin leads to induction of Noxa.

## Conclusions

Our study shows that the benzimidazoles potentiate navitoclax activity through induction of Noxa. These data suggest that Noxa-inducing agents would be good candidates to combine with navitoclax, consistent with other studies [33-35,41]. In the future, *in vivo* studies should be performed to validate our *in vitro* findings. However, other benzimidazoles with better pharmacokinetics would be required since albendazole and mebendazole are rapidly metabolized in the liver [30]. In addition, biomarker identification for patient selection for such combination would dictate the success of these findings. Nevertheless, the methods we report here are broadly applicable for identification of new combination therapies and novel mechanisms.

## Materials and methods

### Reagents

Navitoclax was synthesized at AbbVie, Inc. (North Chicago, IL). FDA-approved drug library was purchased from Enzo (Farmingdale, NY). Albendazole, mebendazole, oxibendazole, and oxfendazole were purchased from Sigma (St. Louis, MO). All siRNA pools were purchased from Dharmacon (Lafayette, CO). Noxa antibody was purchased from Abcam. Bim and Mcl-1 antibodies were purchased from Epitomics (Burlingame, CA). Caspase 3 and caspase 9 inhibitors were purchased from SantaCruz Biotechnology (Santa Cruz, CA). All the branched DNA reagents were purchased from Affymetrix (Santa Clara, CA).

### Cell culture, transfection, and cell-based assays

All NSCLC cell lines (ATCC, Manassas, VA) were cultured in RPMI (Invitrogen Corp., Grand Island, NY) supplemented with 10% fetal bovine serum (Invitrogen), 1% sodium pyruvate, 4.5 g/L glucose, and antibiotics (Invitrogen) and were maintained in a humidified chamber at 37°C containing 5% CO_2_. These cells were authenticated by morphologic, cell proliferation, and Mycoplasma tests recommended in the ATCC Technical Bulletin No. 8 (2007).

siRNAs were introduced into the cells by reverse transfection using Lipofectamine2000 according to manufacturer’s instructions (Invitrogen). Briefly, siRNAs were first mixed with Lipofectamine2000 in Opti-MEM (Invitrogen). Cells were added at 1.5 − 2.5 × 10^4 cells/ 100 μl in 96-well tissue culture plates after 15 minutes. A final concentration of 20 nM siRNA was used. The cells were then grown in medium without antibiotic for 1 day before adding navitoclax. Forty-eight hours after transfection, cells were assayed for viability using CellTiter Glo Luminescent cell viability assay according to the manufacturer’s protocol (Promega, Madison, WI). Statistical analysis was carried out using Microsoft Excel to determine p value (2-tailed) and p < 0.05 was indicated by * in the figures.

### Western blot analysis

Cell lysates were prepared in RIPA buffer (Sigma) with protease inhibitor cocktail (Roche). 30 μg of total protein was resolved on a 12% SDS polyacrylamide gel and probed with anti-Bim, anti-Noxa and anti-Mcl-1. Antibody against actin (Santa Cruz Biotechnology, Santa Cruz, CA) was used as a loading control.

### Branched DNA assay

Cell lysates were prepared in lysis mixture (Affymetrix, CA) with proteinase K (Affymetrix, CA). Branched DNA assay for Noxa, Bim, Puma, Mcl-1, Bcl-x_L_, and HPRT was designed by Affymetrix and was performed according to the manufacturer’s instruction. The expression of these genes was presented after normalizing to HPRT expression and no treatment control.

### Cytotoxicity assay screen of the FDA approved small molecule library

Hcc827 cells were plated overnight before adding the compounds. Medium was added to the compound library immediately before adding to the cells. The cells with or without navitoclax treatment were added simultaneously or sequentially. NSCLC cell line Hcc827 was screened with four concentrations (0.78, 3.13, 12.5, 50 μM) of the FDA approved compounds in the presence or absence of 1 μM navitoclax for 1 or 3 days. Cells were assayed for viability using CellTiter Glo Luminescent cell viability assay according to the manufacturer’s protocol (Promega, Madison, WI).

Further validation was performed in three NSCLC cell lines with increasing concentration of benzimidazoles and navitoclax. Synergistic activities of navitoclax and benzimidazoles were determined using the Bliss additivity model [44], whereby the combined response C of both agents with individual effects A and B is C = A + B – (A × B), where A and B represent the fractional inhibition between 0 and 1. Combined response scores greater than 15 were considered synergistic; 0–15 were considered no interaction, and less than −15 were considered antagonistic.

## Additional files

Additional file 1: Figure S1. Multiple antihelminthic benzimidazoles potentiate navitoclax activity in H292 cells. H292 cells were treated with increasing concentrations of four different benzimidazoles in the presence or absence of 1 μM navitoclax. Viability was determined after 1 day. Additional file 2: Figure S2. Levels of knockdown by Bim and Noxa siRNA in Hcc827 and H292 cells. Hcc827 and H292 cells were transfected with Noxa or Bim or control siRNA for 2 days. Cell lysate was prepared and resolved on a 12% SDS polyacrylamide gel and probed with anti-Bim, anti-Noxa, and anti-Mcl-1. Antibody against actin was used as a loading control. Additional file 3: Figure S3. Antihelminthic compound albendazole potentiates navitoclax activity in MDA-MB-231, Hcc1806, Ovcar4, and PC3 cells. MDA-MB-231, Hcc1806, Ovcar4, and PC3 cells were treated with increasing concentrations of albendazole in the presence or absence of 1 μM navitoclax. Viability was determined after 1 day. Additional file 4: Figure S4. No correlation between TP53 mutation status and mebendazole sensitivity in a panel of NSCLC cell lines.

## Abbreviations

NSCLC: Non-small cell lung cancer
ER: Endoplasmic reticulum
ERAD: ER-associated protein degradation
