# Supplementary figures and images for: Antihelminthic benzimidazoles potentiate navitoclax (ABT-263) activity by inducing Noxa-dependent apoptosis in non-small cell lung cancer (NSCLC) cell lines
Source: Cancer Cell Int. 2015 Feb 4;15(1):5. doi: 10.1186/s12935-014-0151-3 (PMC4326508; doi:10.1186/s12935-014-0151-3)

## Slide 1
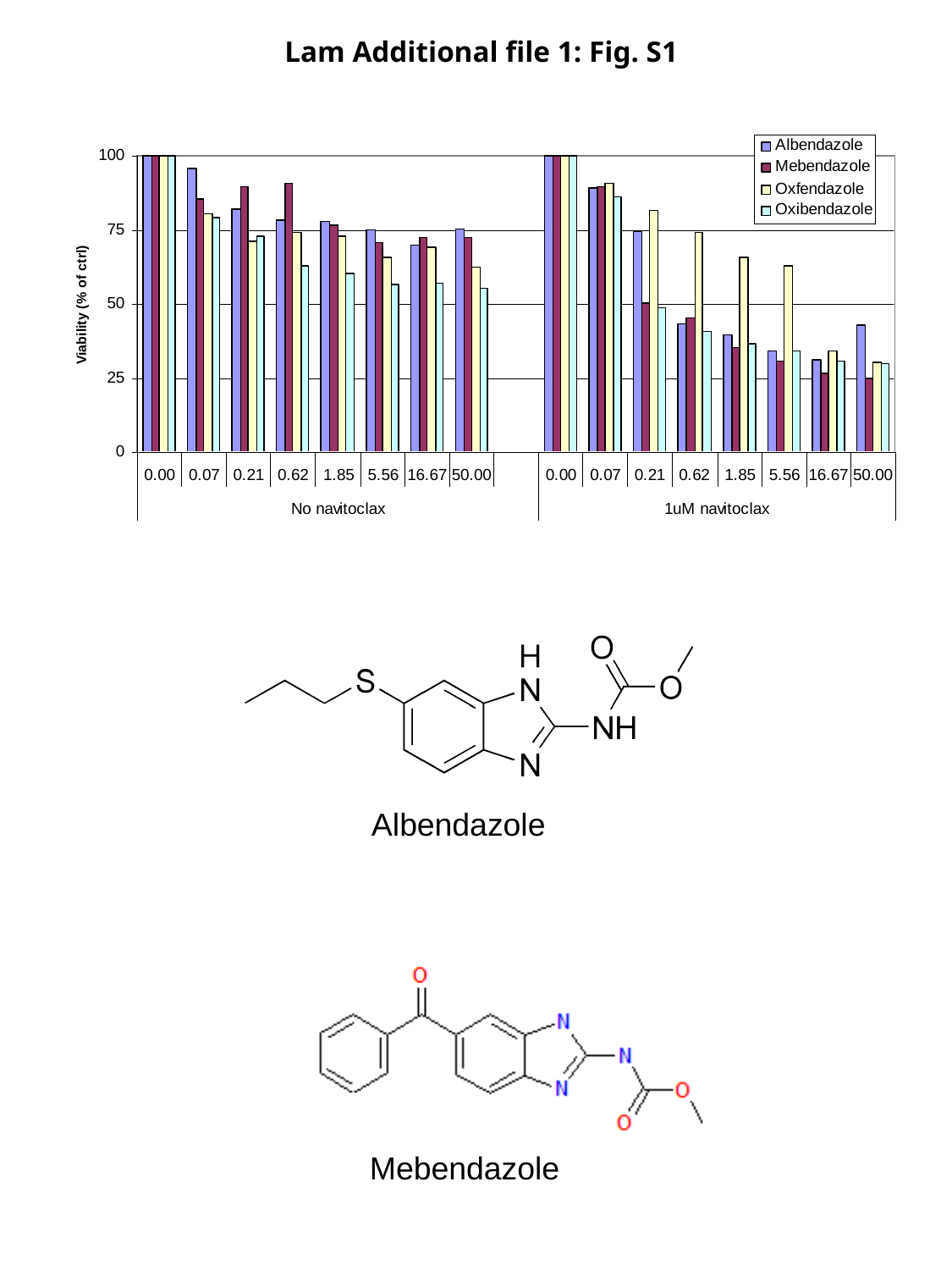

Lam Additional file 1: Fig. S1
Albendazole
Mebendazole

Supplement: Additional file 1: Figure S1. — Multiple antihelminthic benzimidazoles potentiate navitoclax activity in H292 cells. H292 cells were treated with increasing concentrations of four different benzimidazoles in the presence or absence of 1 μM navitoclax. Viability was determined after 1 day. [file 12935_2014_151_MOESM1_ESM.pptx]

## Slide 1
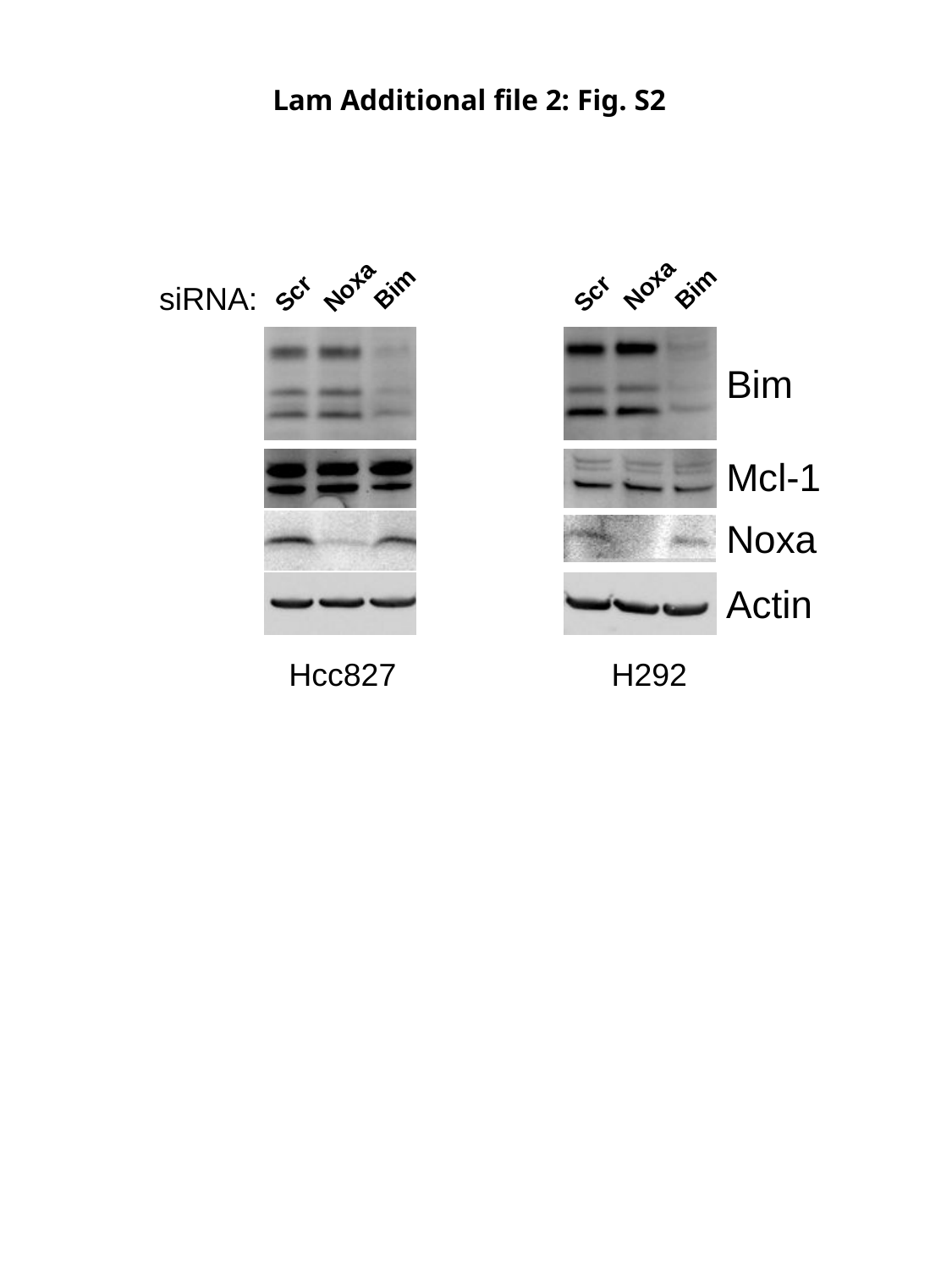

Lam Additional file 2: Fig. S2
Bim
Bim
Noxa
Scr
Noxa
Scr
siRNA:
Bim
Mcl-1
Noxa
Actin
H292
Hcc827

Supplement: Additional file 2: Figure S2. — Levels of knockdown by Bim and Noxa siRNA in Hcc827 and H292 cells. Hcc827 and H292 cells were transfected with Noxa or Bim or control siRNA for 2 days. Cell lysate was prepared and resolved on a 12% SDS polyacrylamide gel and probed with anti-Bim, anti-Noxa, and anti-Mcl-1. Antibody against actin was used as a loading control. [file 12935_2014_151_MOESM2_ESM.pptx]

## Slide 1
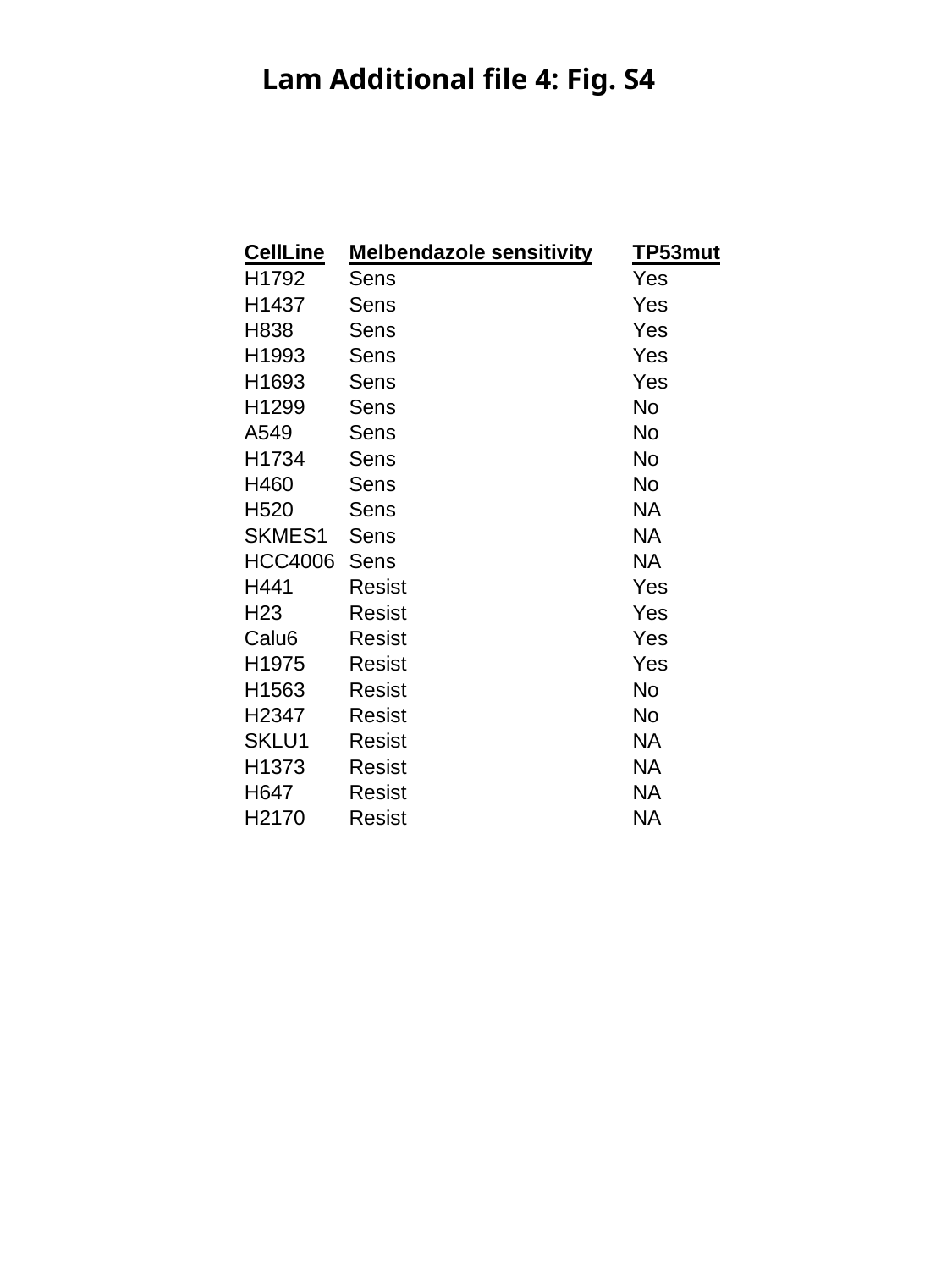

Lam Additional file 4: Fig. S4

Supplement: Additional file 4: Figure S4. — No correlation between TP53 mutation status and mebendazole sensitivity in a panel of NSCLC cell lines. [file 12935_2014_151_MOESM4_ESM.pptx]
