# Supplementary material for: Antihelminthic benzimidazoles potentiate navitoclax (ABT-263) activity by inducing Noxa-dependent apoptosis in non-small cell lung cancer (NSCLC) cell lines
Source: Cancer Cell Int. 2015 Feb 4;15(1):5. doi: 10.1186/s12935-014-0151-3 (PMC4326508; doi:10.1186/s12935-014-0151-3)
Supplement: Additional file 3: Figure S3. — Antihelminthic compound albendazole potentiates navitoclax activity in MDA-MB-231, Hcc1806, Ovcar4, and PC3 cells. MDA-MB-231, Hcc1806, Ovcar4, and PC3 cells were treated with increasing concentrations of albendazole in the presence or absence of 1 μM navitoclax. Viability was determined after 1 day. [file 12935_2014_151_MOESM3_ESM.pptx]

## Slide 1
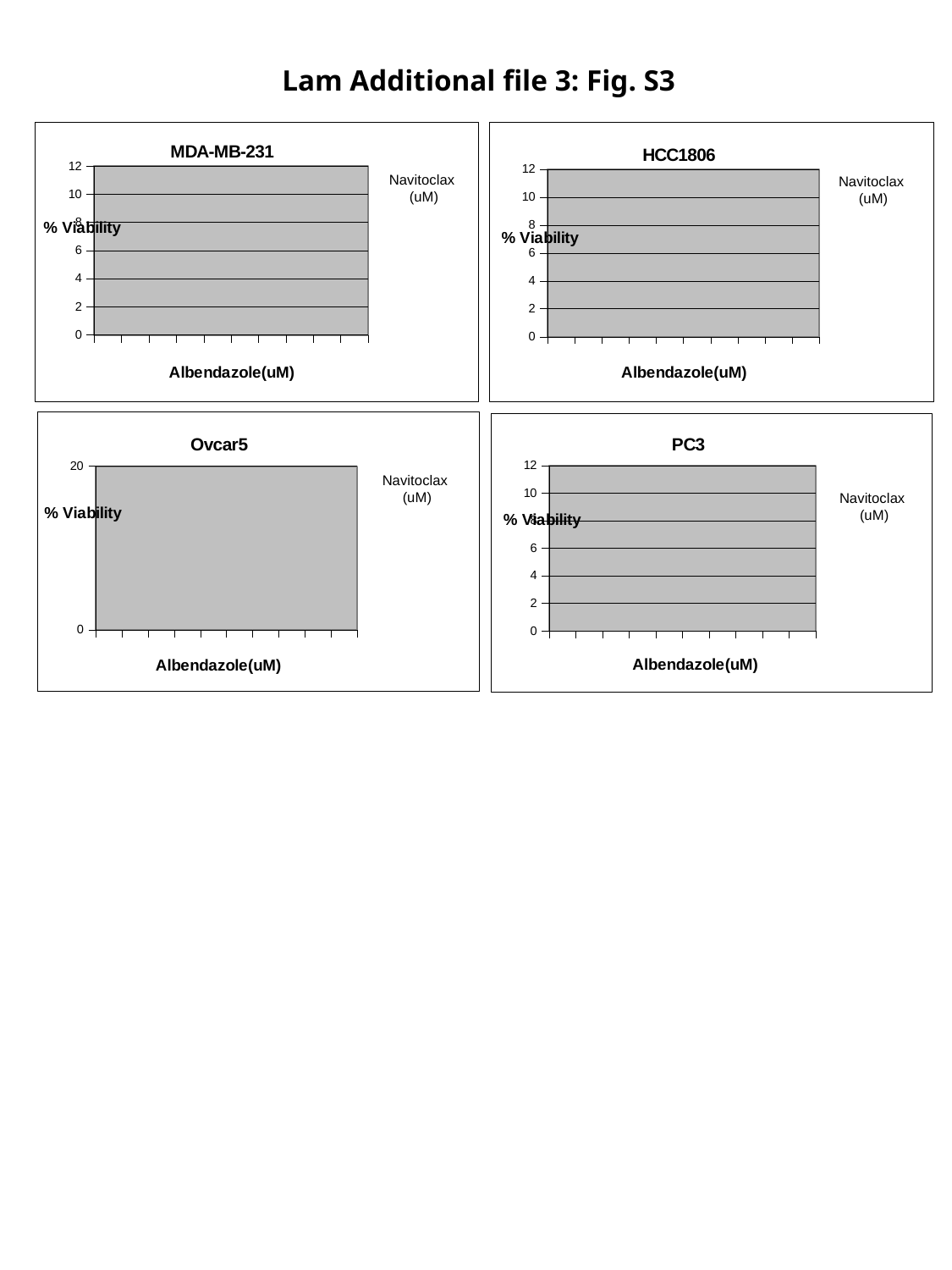

Lam Additional file 3: Fig. S3
### Chart: HCC1806
| Category | | | | |
|---|---|---|---|---|
### Chart: MDA-MB-231
| Category | | | | |
|---|---|---|---|---|Navitoclax
 (uM)
Navitoclax
 (uM)
### Chart: Ovcar5
| Category | | | | |
|---|---|---|---|---|
### Chart: PC3
| Category | | | | |
|---|---|---|---|---|Navitoclax
 (uM)
Navitoclax
 (uM)
